# Investigation of Lipoproteins Oxidation Mechanisms by the Analysis of Lipid Hydroperoxide Isomers

**DOI:** 10.3390/antiox10101598

**Published:** 2021-10-12

**Authors:** Shunji Kato, Yusuke Osuka, Saoussane Khalifa, Takashi Obama, Hiroyuki Itabe, Kiyotaka Nakagawa

**Affiliations:** 1 Food and Biodynamic Chemistry Laboratory, Graduate School of Agricultural Science, Tohoku University, Sendai 980-0845, Miyagi, Japan; shunji.kato.b5@tohoku.ac.jp (S.K.); y_osuka@calbee.co.jp (Y.O.); khalifa.saoussane.q3@dc.tohoku.ac.jp (S.K.); 2 Division of Biological Chemistry, Department of Pharmaceutical Sciences, Showa University School of Pharmacy, 1-5-8 Hatanodai, Shinagawa-ku, Tokyo 142-8555, Japan; obama@pharm.showa-u.ac.jp (T.O.); h-itabe@pharm.showa-u.ac.jp (H.I.)

**Keywords:** lipid hydroperoxide isomers, lipoproteins, mass spectrometry, oxidative stress

## Abstract

The continuous formation and accumulation of oxidized lipids (e.g., lipid hydroperoxides (LOOH)) which are present even in plasma lipoproteins of healthy subjects, are ultimately considered to be linked to various diseases. Because lipid peroxidation mechanisms (i.e., radical, singlet oxygen, and enzymatic oxidation) can be suppressed by certain proper antioxidants (e.g., radical oxidation is efficiently suppressed by tocopherol), in order to suppress lipid peroxidation successfully, the determination of the peroxidation mechanism involved in the formation of LOOH is deemed crucial. In this study, to determine the peroxidation mechanisms of plasma lipoproteins of healthy subjects, we develop novel analytical methods using liquid chromatography-tandem mass spectrometry (LC-MS/MS) for 1-palmitoyl-2-linoleoyl-*sn*-glycero-3-phosphocholine hydroperoxide (PC 16:0/18:2;OOH) and cholesteryl linoleate hydroperoxide (CE 18:2;OOH) isomers. Using the newly developed methods, these PC 16:0/18:2;OOH and CE 18:2;OOH isomers in the low-density lipoprotein (LDL) and high-density lipoprotein (HDL) of healthy subjects are analyzed. Consequently, it is found that predominant PC 16:0/18:2;OOH and CE 18:2;OOH isomers in LDL and HDL are PC 16:0/18:2;9OOH, PC 16:0/18:2;13OOH, CE 18:2;9OOH, and CE 18:2;13OOH, which means that PC and CE in LDL and HDL are mainly oxidized by radical and/or enzymatic oxidation. In conclusion, the insights about the oxidation mechanisms shown in this study would be useful for a more effective suppression of oxidative stress in the human organism.

## 1. Introduction

**Figure 1:**
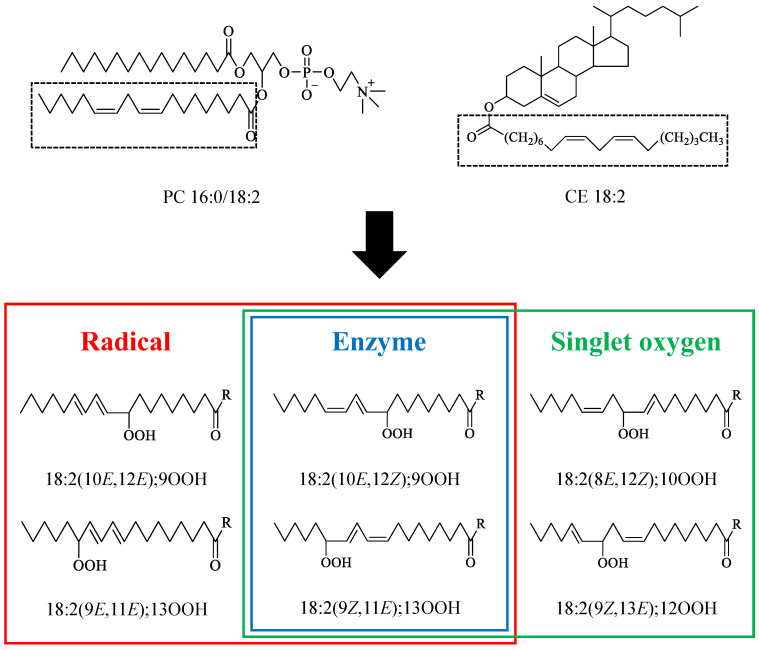
1-Palmitoyl-2-linoleoyl-*sn*-glycero-3-phosphocholine (PC 16:0/18:2) and cholesteryl linoleate (CE 18:2) peroxidation mechanisms and the structures of their hydroperoxide isomers. PC 16:0/18:2 and CE 18:2 are oxidized to 1-palmitoyl-2-linoleoyl-*sn*-glycero-3-phosphocholine hydroperoxide (PC 16:0/18:2;OOH) and cholesteryl linoleate hydroperoxide (CE 18:2;OOH) isomers, respectively, by radical, enzymatic, and singlet oxygen (^1^O_2_) oxidation. Hydroperoxyl group position and geometrical structure depend on the peroxidation mechanisms. The shorthand notation of lipids was in accordance with LIPID MAPS (Liebisch, G. et al. *J. Lipid Res.* **2020**, *61*, 1539–1555 [8]), e.g., PC 16:0/18:2(10*E*,12*E*);9OOH means 1-palmitoyl-2-(9-hydroperoxy-10*E*,12*E*-octadecadienoyl)-*sn*-glycero-3-phosphocholine.

Since oxidized lipids (e.g., primary oxidation products, lipid hydroperoxides (LOOH)) are detected even in a healthy organism, such as in human plasma lipoproteins [1,2,3,4,5], lipid peroxidation certainly proceeds at early stages before illness. The continuous formation and accumulation of LOOH are, ultimately, considered to be linked to various diseases (e.g., atherosclerosis [5], diabetes [4], and Alzheimer’s disease [6]) and an accelerated senescence [1]. Thus, it is of uttermost importance to fight against lipid peroxidation at early stages before the occurrence of any health impairments. This issue has been gaining considerable attention as it can be demonstrated in the expansion of antioxidants usage in our daily life (e.g., supplements and additives). However, it is necessary to note that an antioxidant does not always effectively prevent the formation of LOOH in plasma lipoproteins. As a matter of fact, lipid peroxidation proceeds to form LOOH following three different mechanisms (i.e., radical, singlet oxygen (^1^O_2_), and enzymatic oxidation (e.g., lipoxygenases)), yielding each, different LOOH isomers (Figure 1). Because each mechanism can be suppressed by certain proper antioxidants (e.g., radical oxidation is efficiently suppressed by tocopherol [7]), in order to suppress lipid peroxidation successfully, the determination of the oxidation mechanism involved in the formation of LOOH, especially at early stages, is deemed crucial.

As already mentioned, the isomerism (i.e., hydroperoxyl group position) of LOOH is decided based on the oxidation mechanism involved (Figure 1). This fact suggests that the analysis of LOOH isomers can provide insights about the oxidation mechanisms that take place in our body [7,9]. We recently developed unique mass spectrometric methods using a sodium ion for the analysis of LOOH isomers [5,10,11]. By using these methods, we focused on one of the major LOOH (i.e., 1-palmitoyl-2-linoleoyl-*sn*-glycero-3-phosphocholine hydroperoxide (PC 16:0/18:2;OOH)) in human plasma, determined some isomers of PC 16:0/18:2;OOH (i.e., PC 16:0/18:2;9OOH and PC 16:0/18:2;13OOH), and reported that PC 16:0/18:2;9OOH and PC 16:0/18:2;13OOH would be major isomers of PC 16:0/18:2;OOH in human plasma [5]. In other words, other isomers (i.e., PC 16:0/18:2;10OOH and PC 16:0/18:2;12OOH) that are formed solely by ^1^O_2_ oxidation were assumed to be low in the plasma. Taken together, we hypothesized that the LOOH present in healthy human plasma lipoproteins are mainly derived from radical and/or enzymatic oxidation, rather than ^1^O_2_ oxidation. To prove this hypothesis, the analysis of PC 16:0/18:2;10OOH and PC 16:0/18:2;12OOH in addition to PC 16:0/18:2;9OOH and PC 16:0/18:2;13OOH in plasma lipoproteins is essential. On top of that, because cholesterol ester (CE), which is another major lipid class of plasma lipoproteins, is also prone to oxidation, the analysis of cholesteryl linoleate hydroperoxide (CE 18:2;OOH) isomers will provide further evidence to confirm our speculation.

From these circumstances, in this study, in order to prove the proposed hypothesis (i.e., LOOH present in lipoproteins of healthy subjects is mainly derived from radical and/or enzymatic oxidation), we develop novel analytical methods for all the possible PC 16:0/18:2;OOH isomers and CE 18:2;OOH isomers: PC 16:0/18:2(10*E*,12*E*);9OOH, PC 16:0/18:2(10*E*,12*Z*);9OOH, PC 16:0/18:2(8*E*,12*Z*);10OOH, PC 16:0/18:2(9*E*,11*E*);13OOH, PC 16:0/18:2(9*Z*,11*E*);13OOH, PC 16:0/18:2(9*Z*,13*E*);12OOH, CE 18:2(10*E*,12*E*);9OOH, CE 18:2(10*E*,12*Z*);9OOH, CE 18:2(8*E*,12*Z*);10OOH, CE 18:2(9*E*,11*E*);13OOH, CE 18:2(9*Z*,11*E*);13OOH, and CE 18:2(9*Z*,13*E*);12OOH (Figure 1). Authentic references of these LOOH are prepared and analyzed using liquid chromatography-tandem mass spectrometry (LC-MS/MS). The analyses are performed in each of the lipoprotein classes (chylomicron (CM), very-low-density lipoprotein (VLDL), low-density lipoprotein (LDL), and high-density lipoprotein (HDL)) to investigate whether there are differences between the oxidation mechanisms of individual lipoproteins. The insights obtained in this study will provide a better understanding of the choice of antioxidants to be incorporated in our daily life, based on the oxidation mechanism taking place in our body.

## 2. Materials and Methods

### 2.1. Materials

Linoleic acid (FA 18:2), cholesterol, butylhydroxytoluene (BHT), and 2-methoxypropene (MxP) were purchased from FUJIFILM Wako Pure Chemical Corporation (Osaka, Japan). Cholesteryl linoleate (CE 18:2) was obtained from Tokyo Chemical Industry Co., Ltd. (Tokyo, Japan). 1-Palmitoyl-2-linoleoyl-*sn*-glycero-3-phosphocholine (PC 16:0/18:2) and 1-palmitoyl-2-hydroxy-*sn*-glycero-3-phosphocholine (LPC 16:0/0:0) were from Avanti Polar Lipids (Alabaster, AL, USA). All other reagents were of the highest grade available.

### 2.2. Preparation of PC 16:0/18:2;OOH and CE 18:2;OOH Isomers Standards

In the preparation of PC 16:0/18:2;OOH and CE 18:2;OOH isomers, linoleic acid hydroperoxide (FA 18:2;OOH) isomers (FA 18:2(10*E*,12*Z*);9OOH, FA 18:2(10*E*,12*E*);9OOH, FA 18:2(8*E*,12*Z*);10OOH, FA 18:2(9*Z*,13*E*);12OOH, FA 18:2(9*Z*,11*E*);13OOH, and FA 18:2(9*E*,11*E*);13OOH) were firstly prepared following our previous report [5,10]. The hydroperoxyl group of the resulting FA 18:2;OOH isomers was then protected with MxP for subsequent esterification [12]. Protected FA 18:2;OOH isomers were esterified with LPC 16:0/0:0 or cholesterol [12,13]. Protected PC 16:0/18:2;OOH and CE 18:2;OOH isomers were finally deprotected and purified as in a previous study [12,13].

The concentration of the standard of PC 16:0/18:2;OOH isomers was determined by quantifying inorganic phosphorus according to Bartlett method [14]. To determine the concentration of CE 18:2;OOH isomers, HPLC with chemiluminescence detection was used [1]. The separation was achieved using a COSMOSIL 5C_18_-MS-II column (5.0 µm, 4.6 × 250 mm, nacalai tesque, INC., Kyoto, Japan) with methanol/2-propanol (3:2, *v*/*v*) as the mobile phase (1 mL/min) at 40 °C. The concentration of CE 18:2;OOH isomers was calculated by using the standard of triacylglycerol hydroperoxide, whose concentration was known [15].

### 2.3. MS/MS and LC-MS/MS Analyses of PC 16:0/18:2;OOH and CE 18:2;OOH Isomers

For Q1 and product ion mass spectra, prepared PC 16:0/18:2;OOH and CE 18:2;OOH isomers (1 µM in methanol) were directly infused to a 4000QTRAP quadrupole/linear ion-trap tandem mass spectrometer (SCIEX, Tokyo, Japan) with electrospray ionization (ESI) at a flow rate (10 μL/min). For subsequent multiple reaction monitoring (MRM) analysis, optimum precursor to product ion transitions was set by Analyst^®^ 1.6.2 software (Supplementary Table S1).

LC-MS/MS analyses of PC 16:0/18:2;OOH and CE 18:2;OOH isomers were carried out with ExionLC HPLC/UHPLC system (SCIEX, Tokyo, Japan) attached to a 4000 QTRAP. For the analysis of PC 16:0/18:2;OOH isomers, a COSMOSIL 5C_18_-MS-II column (2.5 µm, 2.0 × 150 mm, nacalai tesque, INC., Kyoto, Japan) was used with a binary gradient consisting of solvent A (water) and solvent B (methanol). The gradient profile was as follows: 0–16 min; 89–97.8% B, 16–16.1 min; 97.8–100% B, 16.1–26 min; 100% B, 26–26.1 min; 100–89% B, 26.1–29.5 min; 89% B. The flow rate gradient was set at 0–16.0 min; 0.2 mL/min, 16.0–16.1 min; 0.2–0.3 mL/min; 16.1–26.0 min; 0.3–0.4 mL/min, 26.1–29.5 min; 0.4–0.2 mL; the column temperature was maintained at 40 °C. The eluent was mixed with a post-column solvent consisting of methanol containing 2 mM sodium acetate at 0.01 mL/min. The analysis of CE 18:2;OOH isomers was performed by using an Inertsil SIL-100A column (5 µm, 2.1 × 250 mm, GL Sciences Inc., Tokyo, Japan). Hexane/2-propanol/acetic acid (27,424:100:1, *v*/*v*/*v*) was used as a mobile phase (isocratic mode) at 40 °C. The flow rate gradient was set at 0–8 min; 0.4–0.2 mL/min, 8–25 min; 0.2 mL/min, 25–30 min; 0.2–0.4 mL/min. The eluent was mixed with a post-column solvent consisting of 2-propanol/methanol (1:1, *v*/*v*) containing 0.2 mM sodium acetate at 0.2 mL/min. PC 16:0/18:2;OOH and CE 18:2;OOH isomers were detected using the MRM constructed above (Supplementary Table S1), and external standard curves were individually established.

### 2.4. LC-MS Analysis of PC 16:0/18:2 and CE 18:2

To analyze PC 16:0/18:2 and CE 18:2, a Nexera UHPLC/HPLC system (Shimazu, Kyoto, Japan) equipped with a micrOTOF-Q II (Bruker Daltonics, GmbH, Bremen, Germany) was used. The detections were performed by Q1 mass scan mode (*m/z* 780.5 ± 0.1 ([M+Na]^+^) for PC 16:0/18:2 and *m/z* 671.5 ± 0.1 ([M+Na]^+^) for CE 18:2). Instrumental MS parameters were optimized by OTOF control Ver. 3.2 (Bruker Daltonics, GmbH, Bremen, Germany) (Supplementary Table S2). Both PC 16:0/18:2 and CE 18:2 were separated by a COSMOSIL 5C_18_-MS-II column (2.5 µm, 2.0 × 150 mm, nacalai tesque, INC., Kyoto, Japan) at 40 °C. For the analysis of PC 16:0/18:2, mobile phases A (water) and B (methanol) were used with a binary gradient as follows: 0–12 min; 90–100% B, 12–30 min; 100% B, 30–30.1 min; 100–90% B, 30.1–34.9 min; 90% B. Additionally, the flow rate gradient was set at 0–12.0 min; 0.3–0.2 mL/min, 12.0–30.0 min; 0.2 mL/min, 30.0–30.1 min; 0.2–0.3 mL/min, 30.1–34.9 min; 0.3 mL/min. On the other hand, for the analysis of CE 18:2, mobile phases A (methanol) and B (2-propanol) were used with a binary gradient as follows: 0–5 min; 25–30% B, 5–10 min; 30–40% B, 10–20 min; 40–70% B, 20–25 min; 70% B, 25–25.1 min; 70–25% B, 25.1–30.1 min; 25% B. Flow rate was set at 0.2 mL/min. Concentration of PC 16:0/18:2 and CE 18:2 was calculated by using external standard curves.

### 2.5. Preparation of Healthy Human Plasma and Lipoproteins

Blood samples were collected from 9 healthy subjects (4 men and 5 women, mean age 25 ± 4 years). The subjects were non-obese and had no diagnosed abnormality of the liver by health check. Additionally, they did not have any dietary restrictions. Participants ate bread (100 g) with butter (8 g) after 12 h of fasting. Three hours after the meal, blood samples were intravenously collected with ethylenediaminetetraacetic acid (EDTA). Obtained blood was centrifuged (1000× *g* for 10 min at 4 °C) to prepare plasma samples. Immediately, using the plasma (5.4 mL), the preparation of lipoproteins (CM, VLDL, LDL, and HDL) was performed as in a previous report with little modification [16]. PBS (including 250 mM EDTA, pH 7.4 (5.4 µL)) was mixed with the plasma. On top of the mixture, PBS (including 250 µM EDTA, pH 7.4 (1.8 mL)) was layered and the sample was centrifuged at 36,700× *g* for 30 min at 4 °C (Optima L -100 XP, Beckman Coulter, Inc., Brea, CA, USA) with a rotor (Type70.1Ti, Beckman Coulter, Inc., Brea, CA, USA). Upper layer (900 µL) was corrected as a CM fraction. Top 900 µL of the remainder was discarded to remove residual CM. Subsequently, PBS (including 250 µM EDTA, pH 7.4 (1.8 mL)) was layered and the sample was centrifuged at 370,500× *g* for 4.05 h at 4 °C. Upper layer (1080 µL) was corrected as a VLDL fraction. Top 720 µL of the remainder was discarded to remove residual VLDL. The remainder was mixed with potassium bromide aq. (50% (*w*/*v*), 1080 µL) to adjust the density to 1.063 g/mL. After centrifugation (370,500× *g* for 4.05 h at 4 °C), upper layer (1080 µL) was corrected as a LDL fraction. Top 1980 µL of the solution was discarded to remove residual LDL. Finally, the remainder was mixed with potassium bromide aq. (50% (*w*/*v*), 3574 µL) to adjust the density to 1.210 g/mL. After centrifugation (370,500× *g* for 6.46 h at 4 °C), upper layer (900 µL) was corrected as a HDL fraction. LDL and HDL fractions were dialyzed against PBS containing 250 µM EDTA. The purity of each lipoprotein was checked by agarose gel electrophoresis. Each lipoprotein sample was stored at −80 °C until the extraction.

This study was approved by the research ethics committee (17-A-01 and 18-A-03) of Tohoku University (Sendai, Japan), and informed consent was obtained from all participants.

### 2.6. Extraction of PC 16:0/18:2;OOH (PC 16:0/18:2) and CE 18:2;OOH (CE 18:2)

PC 16:0/18:2;OOH (PC 16:0/18:2) and CE 18:2;OOH (CE 18:2) were extracted by modified Folch method [5,17]. Each lipoprotein solution (350 µL) was mixed with 250 µL 0.9% KCl (containing 1 mM EDTA) aqueous solution and 2.4 mL chloroform/methanol (2:1, *v*/*v*, 0.002% BHT). After mixing (5 min, 2500 rpm), the solution was centrifuged at 1660× *g* for 20 min at 4 °C. The lower layer (lipid fraction) was collected, and upper layer was re-extracted with 1.4 mL chloroform/methanol (10:1, *v*/*v*). After mixing (1 min, 2500 rpm), the solution was centrifuged as above. For the subsequent solid phase extraction (SPE), the combined lower layers were mixed with 2-propanol/hexane (3:7, *v*/*v*), and loaded onto an SPE column (Strata NH_2_, 1 mL, 100 mg, Phenomenex, Torrance, CA, USA) equilibrated with chloroform. Elution was collected as a crude CE 18:2;OOH (CE 18:2) fraction. The column was further eluted with 2 mL of chloroform, and the elution was also combined to the previous crude CE 18:2;OOH (CE 18:2) fraction. Subsequently, PC 16:0/18:2;OOH (PC 16:0/18:2) fraction was eluted by 2 mL methanol from the column. PC 16:0/18:2;OOH (PC 16:0/18:2) fraction was evaporated under N_2_ gas, and the residue was dissolved in 525 µL methanol. The obtained crude CE 18:2;OOH (CE 18:2) fraction was evaporated under N_2_ gas then dissolved in hexane for further purification. The crude CE 18:2;OOH (CE 18:2) fraction was then loaded onto an SPE column (Strata NH_2_, 1 mL, 100 mg) which was equilibrated with hexane (3 mL). Elution was collected as a CE 18:2;OOH (CE 18:2) fraction. The column was further eluted with 2 mL of hexane/diethyl ether (1:1, *v*/*v*), and the elution was also combined to the previous CE 18:2;OOH (CE 18:2) fraction. A fraction of the CE 18:2;OOH (CE 18:2) was evaporated under N_2_ gas, and the residue was dissolved in 525 µL hexane.

### 2.7. Statistics

In this study, limits of quantification (LOQ) were set as follows: 0.075 pmol for PC 16:0/18:2(10*E*,12*Z*);9OOH, 0.075 pmol for PC 16:0/18:2(10*E*,12*E*);9OOH, 0.0075 pmol for PC 16:0/18:2;10OOH, 0.0075 pmol for PC 16:0/18:2;12OOH, 0.015 pmol for PC 16:0/18:2;13OOH, 0.033 pmol for CE 18:2;9OOH, 0.005 pmol for CE 18:2;10OOH, 0.005 pmol for CE 18:2;12OOH, 0.017 pmol for CE 18:2(9*Z*,11*E*);13OOH, and 0.017 pmol for CE 18:2(9*E*,11*E*);13OOH. LOQ was the minimum concentration of which the coefficient value was within ± 20% [18], when the standard was analyzed three times. The samples with a value under LOQ were excepted from the calculation of the oxidation ratio. The oxidation ratio was calculated by dividing the concentration of PC 16:0/18:2;OOH (CE 18:2;OOH) isomer by the concentration of PC 16:0/18:2 (CE 18:2).

Data are expressed as means ± SDs. The statistical analyses were performed on the data of which the sample number (*n* = 9) was complete. For comparisons between 2 groups, statistically significant differences were determined by Mann–Whitney’s U-test. Differences were considered significant at *p* < 0.05.

## 3. Results

### 3.1. Development of the Analytical Methods for PC 16:0/18:2;OOH and CE 18:2;OOH Isomers

**Figure 2:**
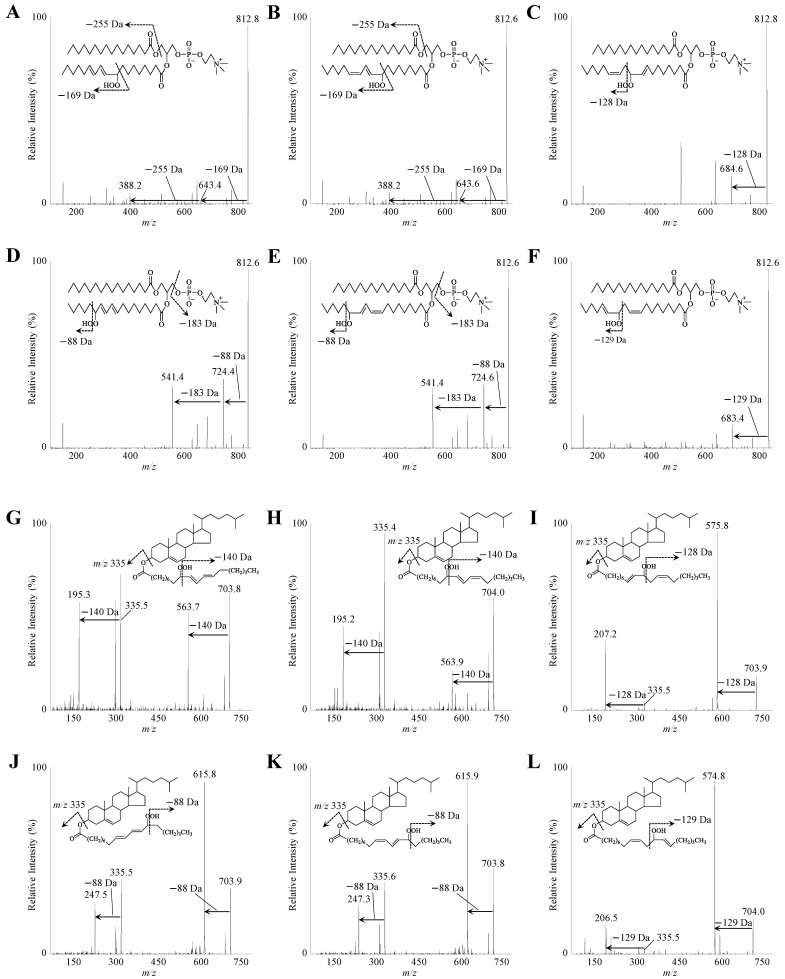
The product ion mass spectra of reference PC 16:0/18:2;OOH (**A**–**F**) and CE 18:2;OOH isomers (**G**–**L**). Reference PC 16:0/18:2;OOH and CE 18:2;OOH isomers were dissolved in methanol (1 µM) and infused directly into the MS/MS system at a flow rate of 10 μL/min. Insets show the speculated fragmentation patterns of PC 16:0/18:2;OOH and CE 18:2;OOH isomers.

First of all, to analyze the structure of the synthesized PC 16:0/18:2;OOH and CE 18:2;OOH isomers, Q1 and product ion mass analyses were carried out. When each PC 16:0/18:2;OOH and CE 18:2;OOH isomer was infused to a 4000 QTRAP, the precursor ions *m/z* 813 ([M+Na]^+^) and *m/z* 704 ([M+Na]^+^) were clearly detected, respectively (data not shown). Therefore, the product ion mass analyses were then performed. Sodiated PC 16:0/18:2;OOH isomers (*m/z* 813 ([M+Na]^+^)) provided the hydroperoxyl group position specific product ions, *m/z* 388 for PC 16:0/18:2;9OOH (Figure 2A,B), *m/z* 684 for PC 16:0/18:2;10OOH (Figure 2C), *m/z* 683 for PC 16:0/18:2;12OOH (Figure 2F), and *m/z* 541 for PC 16:0/18:2;13OOH (Figure 2D,E). Similarly, sodiated CE 18:2;OOH isomers (*m/z* 704 ([M+Na]^+^)) also provided specific product ions, *m/z* 195 for CE 18:2;9OOH (Figure 2G,H), *m/z* 576 for CE 18:2;10OOH (Figure 2I), *m/z* 575 for CE 18:2;12OOH (Figure 2L), and *m/z* 247 for CE 18:2;13OOH (Figure 2J,K). Differences were not observed between mass spectra of *cis–trans* isomers (e.g., PC 16:0/18:2(10*E*,12*E*);9OOH (Figure 2A) and PC 16:0/18:2(10*E*,12*Z*);9OOH (Figure 2B)). Using the observed fragment ions, MRM pairs were constructed.

**Figure 3:**
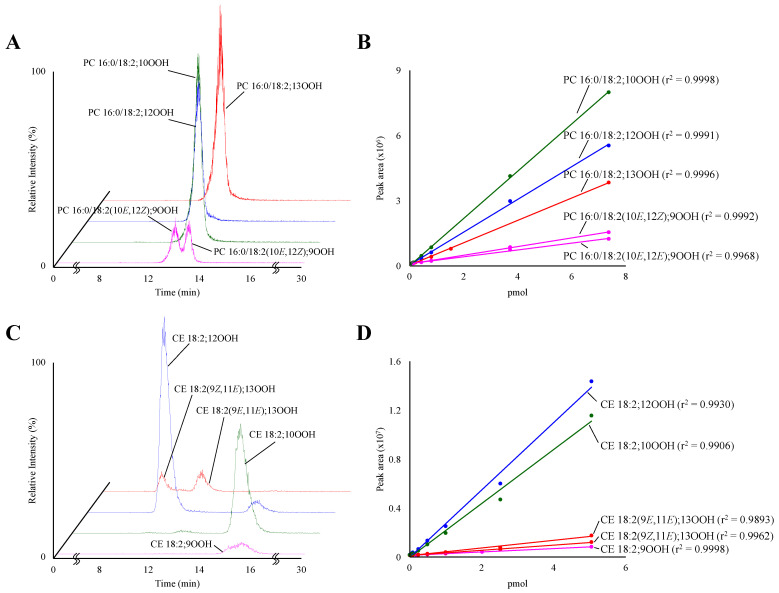
MRM chromatograms of reference PC 16:0/18:2;OOH isomers (**A**) and CE 18:2;OOH isomers (**C**). The references (0.5 pmol each) were analyzed with MRM (*m/z* 813 > 388 for PC 16:0/18:2(10*E*,12*Z*);9OOH and PC 16:0/18:2(10*E*,12*E*);9OOH, *m/z* 813 > 684 for PC 16:0/18:2;10OOH, *m/z* 813 > 683 for PC 16:0/18:2;12OOH, *m/z* 813 > 541 for PC 16:0/18:2;13OOH, *m/z* 704 > 195 for CE 18:2;9OOH, *m/z* 704 > 576 for CE 18:2;10OOH, *m/z* 704 > 575 for CE 18:2;12OOH, and *m/z* 704 > 247 for CE 18:2(9*Z*,11*E*);13OOH and CE 18:2(9*E*,11*E*);13OOH. Calibration curves of reference PC 16:0/18:2;OOH isomers (**B**) and CE 18:2;OOH isomers (**D**).

Using constructed LC-MS/MS conditions, synthesized PC 16:0/18:2;OOH (Figure 3A) and CE 18:2;OOH (Figure 3C) isomers were analyzed. Constructed MRM pairs detected clear peaks. However, it was found that the retention times of PC 16:0/18:2(9*Z*,11*E*);13OOH and PC 16:0/18:2(9*E*,11*E*);13OOH were the same. Therefore, PC 16:0/18:2(9*Z*,11*E*);13OOH and PC 16:0/18:2(9*E*,11*E*);13OOH) were calculated as a total PC 16:0/18:2;13OOH in this study. Similarly, CE 18:2(10*E*,12*Z*);9OOH and CE 18:2(10*E*,12*E*);9OOH were also calculated as a total CE 18:2;9OOH, because these isomers also had the same retention time. Under optimized conditions, calibration curves demonstrated a good linearity for PC 16:0/18:2(10*E*,12*Z*);9OOH (0.075–7.5 pmol, r^2^ = 0.9992), PC 16:0/18:2(10*E*,12*E*);9OOH (0.075–7.5 pmol, r^2^ = 0.9968), PC 16:0/18:2;10OOH (0.0075–7.5 pmol, r^2^ = 0.9998), and PC 16:0/18:2;12OOH (0.0075–7.5 pmol, r^2^ = 0.9991)), PC 16:0/18:2;13OOH (0.015–7.5 pmol, r^2^ = 0.9996) (Figure 3B), CE 18:2;9OOH (0.033–5.00 pmol, r^2^ = 0.9998), CE 18:2;10OOH (0.017–5.0 pmol, r^2^ = 0.9906), CE 18:2;12OOH (0.017–5.0 pmol, r^2^ = 0.9930), CE 18:2(9*Z*,11*E*);13OOH (0.017–5.0 pmol, r^2^ = 0.9962), and CE 18:2(9*E*,11*E*);13OOH (0.017–5.0 pmol, r^2^ = 0.9893) (Figure 3D).

### 3.2. Analysis of PC 16:0/18:2;OOH and CE 18:2;OOH Isomers in Each Lipoprotein of Healthy Subjects

**Figure 4:**
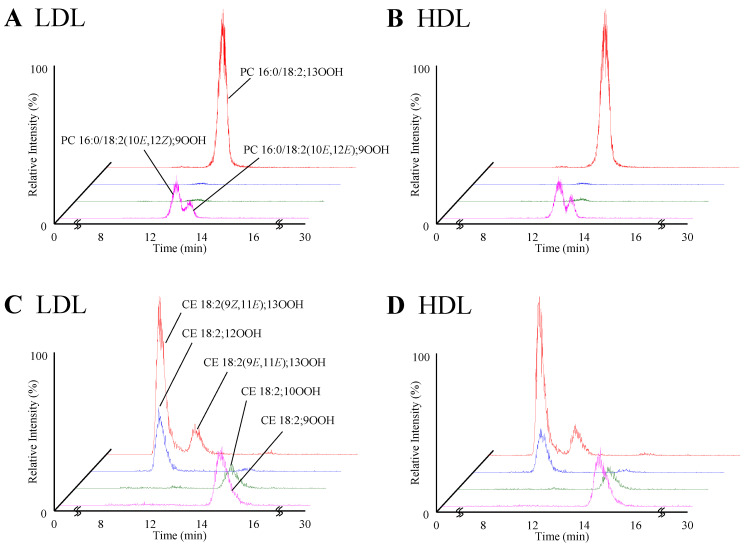
MRM chromatograms of PC 16:0/18:2;OOH isomers (**A**,**B**) and CE 18:2;OOH isomers (**C**,**D**). LOOH extracted from LDL (**A**,**C**) and HDL (**B**,**D**) were analyzed.

When PC 16:0/18:2;OOH and CE 18:2;OOH isomers in each lipoprotein were analyzed, their concentration in CM and VLDL were mostly under LOQ in proportion to the PC 16:0/18:2 and CE 18:2 concentration (Table 1). Therefore, in this study, we focused on the analysis of LDL and HDL oxidation. Regarding the PC 16:0/18:2 oxidation, PC 16:0/18:2;9OOH and PC 16:0/18:2;13OOH were predominantly detected in LDL and HDL (Figure 4A,B). Table 1 shows the ratio of each PC 16:0/18:2;OOH isomer to PC 16:0/18:2 (×10^−3^%). In some samples, the ratio of PC 16:0/18:2;10OOH and PC 16:0/18:2;12OOH could not be calculated due to their low concentration. On the other hand, all CE 18:2;OOH isomers were detected in LDL and HDL (Figure 4C,D). However, such as the PC 16:0/18:2;OOH isomer, the main isomers were CE 18:2;9OOH and CE 18:2;13OOH (Table 1).

The proportion of *cis–trans* isomers was affected by the presence of hydrogen radical donors (e.g., tocopherol) [19]. Thus, to evaluate the effect by antioxidants, the ratios of PC 16:0/18:2(10*E*,12*Z*);9OOH to PC 16:0/18:2(10*E*,12*E*);9OOH and CE 18:2(9*Z*,11*E*);13OOH to CE 18:2(9*E*,11*E*);13OOH were calculated. As a result, the ratio of PC 16:0/18:2(10*E*,12*Z*);9OOH to PC 16:0/18:2(10*E*,12*E*);9OOH was 1.6–2.5, which was remarkably lower than the ratio of CE 18:2(9*Z*,11*E*);13OOH to CE 18:2(9*E*,11*E*);13OOH (5.5–28.1).

The ratio of PC 16:0/18:2(10*E*,12*Z*);9OOH to PC 16:0/18:2 of HDL (71.9 ± 47.1 × 10^−3^%) was significantly higher than that of LDL (32.8 ± 25.8 ×10^−3^%, *p* < 0.05). In addition, the ratio of PC 16:0/18:2;13OOH of HDL (123.6 ± 78.1 × 10^−3^%) also tended to be higher than that of LDL (60.1 ± 47.9 × 10^−3^%, *p* = 0.05). Similarly, the higher oxidation ratio of CE 18:2;OOH of HDL than that of LDL was observed for CE 18:2;9OOH (*p* < 0.05) and CE 18:2(9*E*,11*E*);13OOH (*p* < 0.05).

## 4. Discussion

As described in the introduction, since LOOH are detected even in a healthy organism, such as in human plasma lipoproteins, lipid peroxidation certainly proceeds at early stages before illness. Such a continuous formation and accumulation of LOOH are, ultimately, considered to be linked to various diseases (e.g., 160 ± 65 nM PC;OOH (healthy, *n* = 47) and 331 ± 19 nM (hyperlipidemia, *n* = 94) [1]; ~100 nM PC;OOH (healthy, *n* = 10), ~300 nM (Uremia, *n* = 39), and ~300 nM (hyperlipidemia, *n* = 49) [2]; 33.1 ± 10.2 nM PC 16:0/18:2;9OOH (healthy, *n* = 8), 36.1 ± 11.5 nM PC 16:0/18:2;13OOH (healthy, *n* = 8), 45.2 ± 18.1 nM PC 16:0/18:2;9OOH (patients with angiographically significant stenosis, *n* = 12), and 52.4 ± 24.6 PC 16:0/18:2;13OOH (patients with angiographically significant stenosis, *n* = 12) [5]). Importantly, because each oxidation mechanism (i.e., radical, ^1^O_2_, and enzymatic oxidation, Figure 1) can be suppressed by certain proper antioxidants, in order to suppress lipid peroxidation successfully, the oxidation mechanism involved in the formation of LOOH should be determine. To achieve this, we considered that the analysis of the LOOH isomeric structure in lipoproteins before illness is essential.

With regard to the analysis of lipid oxidation products, it is generally stated that the analysis advances along with the evolution of mass spectrometry [20]. However, even in the use of latest instruments, the analysis of LOOH isomers is a great challenge [21,22,23]. Traditionally, the LOOH isomeric structure is analyzed in the form of fatty acid derivatives after some derivatizations (e.g., reduction, trimethylsilylation, and methanolysis) [24]. To overcome such a complication, Ag^+^ coordination mass spectrometry without derivatization based on Hock fragmentation was previously developed [25,26,27]. This method, however, cannot distinguish half of the hydroperoxide isomers because Hock fragmentation provides the same product ions from the isomers originating from the same double bond (e.g., FA 18:2;9OOH and FA 18:2;10OOH). This means that it is impossible to distinguish radical oxidation from ^1^O_2_ oxidation (Figure 1). Under these circumstances, we discovered that collision-induced dissociation (CID) of sodiated PC 16:0/18:2;OOH provided hydroperoxyl group position specific product ions based on α-cleavage [5]. Using this method, we analyzed PC 16:0/18:2;9OOH and PC 16:0/18:2;13OOH in human plasma and hypothesized the contribution of radical and/or enzymatic oxidation to PC 16:0/18:2;OOH formation in human plasma lipoproteins [5]. Recently, we further showed the evidence that CID of sodiated LOOH is available to various lipid classes, including cholesterol ester [13]. Therefore, in this study, to ensure the above hypothesis (i.e., LOOH present in plasma lipoproteins are mainly derived from radical and/or enzymatic oxidation), we firstly developed novel analytical methods for PC 16:0/18:2;OOH and CE 18:2;OOH isomers.

First of all, to achieve accurate analyses, we prepared authentic standards. When these standards were analyzed, in accordance with our previous studies [5,13], sodiated PC 16:0/18:2;OOH and CE 18:2;OOH isomers generated the hydroperoxyl group position-specific product ions (Figure 2). On the other hand, this study newly showed that sodiated *cis–trans* isomers of PC 16:0/18:2;OOH (e.g., Figure 2A,B) and CE 18:2;OOH (e.g., Figure 2G,H) did not provide the differences in product ions. While we tried to separate these *cis–trans* isomers by HPLC, some *cis–trans* isomers could not be separated. Therefore, PC 16:0/18:2(9*Z*,11*E*);13OOH (CE 18:2(10*E*,12*Z*);9OOH) and PC 16:0/18:2(9*E*,11*E*);13OOH (CE 18:2(10*E*,12*E*);9OOH) were calculated as a total PC 16:0/18:2;13OOH (CE 18:2;9OOH). With regard to the analytical methods by LC-MS/MS, attention should be paid to the matrix effect. On the analysis of oxidized lipids, the matrix effect is mostly induced by co-eluted unoxidized lipids [5]. Therefore, in this study, we constructed LC conditions that PC 16:0/18:2;OOH (CE 18:2;OOH) was completely separated from unoxidized PC (CE). Under the optimum conditions, a good linearity was obtained on each calibration curve of PC 16:0/18:2;OOH and CE 18:2;OOH isomers (Figure 3B,D). Additionally, regarding the LOOH extraction, it is generally stated that LOOH is easily decomposed by certain factors. We previously demonstrated that plasma LOOH was mainly decomposed by contact with water-soluble materials (e.g., metal and salt) [5]. Therefore, to avoid such contact, PC 16:0/18:2;OOH and CE 18:2;OOH were extracted by the modified Folch method and SPE. In this study, we did not use stable isotope standards due to their complex synthetic method. Such stable isotope standards would further upgrade the methods in a future study.

Using the newly developed methods, PC 16:0/18:2;OOH and CE 18:2;OOH were analyzed in CM, VLDL, LDL, and HDL. In this study, PC 16:0/18:2;OOH and CE 18:2;OOH were mostly under LOQ for CM and VLDL. As a reason, it was considered that PC 16:0/18:2 and CE 18:2 concentrations in CM and VLDL solutions prepared in this study were very low (Table 1). On the other hand, PC 16:0/18:2;OOH and CE 18:2;OOH were clearly detected in LDL and HDL (Figure 4). Regarding the PC 16:0/18:2;OOH isomer in LDL and HDL, in agreement with our previous study [5], PC 16:0/18:2;9OOH and PC 16:0/18:2;13OOH were predominantly detected. Additionally, PC 16:0/18:2;10OOH and PC 16:0/18:2;12OOH were mostly not detected, which suggested that PC 16:0/18:2;OOH present in lipoproteins was mainly derived from radical and/or enzymatic oxidation rather than ^1^O_2_ oxidation, as we hypothesized (Table 1). Considering our previous study, that the chirality of PC 16:0/18:2;13OOH was not observed in human plasma [28], the contribution of enzymatic oxidation (at least 15-lipoxygenase) to lipoproteins PC oxidation would not be high in healthy humans. Similar to PC 16:0/18:2;OOH, the main CE 18:2;OOH isomers were CE 18:2;9OOH and CE 18:2;13OOH (Table 1). While CE 18:2;10OOH and CE 18:2;12OOH were detected to some extent compared to PC 16:0/18:2;10OOH and PC 16:0/18:2;12OOH, this would be due to the high concentration of CE 18:2 in LDL and HDL solutions prepared in this study. Therefore, CE 18:2 was also considered to be oxidized mainly by radical and/or enzymatic oxidation.

Regarding the prooxidants which associate with lipoproteins oxidation, some candidates were reported. For instance, in the subendothelial space, NADPH oxidase, xanthine oxidase, myeloperoxidase, and transition metals would contribute to the LDL oxidation [29]. Interestingly, most of them were radical generators, which was consistent with our results stating that LOOH in lipoproteins are mostly radical oxidation products. For reference, we previously reported that PC 16:0/18:2;OOH accumulated in human erythrocyte [6,30]. Considering the fact that LOOH generated in erythrocyte spontaneously migrates to LDL [31], erythrocyte oxidation may also contribute to the LOOH accumulation in lipoproteins. Additionally, it is interesting to note that photo-therapy also induces plasma lipid oxidation [32]. In human plasma, several photo sensitizers are present, and some of these induce type I photo oxidation (i.e., radical oxidation) [33]. These facts may suggest that the contribution of photooxidation to lipoproteins oxidation is quite possible. So far, it is not certain to what extent each prooxidant contributes to lipoproteins oxidation. However, this study showed that the oxidation mechanism of plasma lipoproteins before illness is comprehensively radical oxidation.

Another important observation was that the oxidation ratio of CE was unambiguously lower than that of PC in any of the lipoproteins. Ginger et al. suggested that a lower CE oxidation rate compared with PC was due to the presence of hydrophobic antioxidant (i.e., tocopherol) at the CE-rich hydrophobic core of LDL [19]. To prove this, they analyzed *cis–trans* structures of fatty acid hydro(pero)xide hydrolyzed from PC 16:0/18:2;OOH and CE 18:2;OOH. During lipid peroxidation, the ratio of *cis–trans* to *trans–trans* (e.g., PC 16:0/18:2(10*E*,12*Z*);9OOH to PC 16:0/18:2(10*E*,12*E*);9OOH) depends on the concentration of hydrogen radical donors such as tocopherol [9]. In this study, the ratio of PC 16:0/18:2(10*E*,12*Z*);9OOH to PC 16:0/18:2(10*E*,12*E*);9OOH was 1.6–2.5. On the other hand, the ratio of CE 18:2(9*Z*,11*E*);13OOH to CE 18:2(9*E*,11*E*);13OOH was remarkably higher (5.5–28.1) than that of PC 16:0/18:2;OOH (Table 1). These results suggest the possibility that hydrogen radical donors (e.g., tocopherol) react with the CE radical more effectively than the PC radical. This would be a reason why the oxidation ratio of CE was lower than that of PC.

Furthermore, this study showed a higher oxidation ratio of HDL than that of LDL (especially for PC 16:0/18:2;OOH) (Table 1). A lot of studies reported roughly two antioxidative systems of HDL. One is the detoxification of LOOH, for instance, based on the reduction in LOOH to LOH by Met attributed to apoAI, and the hydrolysis of oxidized fatty acid by PON 1 [34,35]. Another one is the enzymatic correction of LO(O)H in LDL by HDL [36]. These corrected LO(O)H are transported to the liver and metabolized. Summarizing these insights, HDL is considered to play a role for the vehicle of LO(O)H, which might result in the higher oxidation ratio of HDL. This study discussed the oxidation mechanisms focusing on the lipoproteins of healthy humans. Most oxidized lipoproteins would be metabolized in the liver [35,37]. However, the oxidized lipoproteins at a level exceeding the capacity of the metabolism might link to various diseases.

## 5. Conclusions

In summary, to determine the oxidation mechanisms of human plasma lipoproteins before illness, analytical methods for PC 16:0/18:2;OOH and CE 18:2;OOH isomers were developed. To the best of our knowledge, this was the first study which analyzed PC 16:0/18:2;OOH and CE 18:2;OOH isomers (partially including the *cis–trans* isomer) in lipoproteins without any derivatizations. As we hypothesized, it was found that PC and CE in the lipoproteins were mainly oxidized by radical and/or enzymatic oxidation, rather than ^1^O_2_ oxidation. In addition, from the analysis of *cis–trans* isomeric structures, it was considered that CE peroxidation was suppressed more effectively than PC. On the other hand, in this study, the concentrations of PC 16:0/18:2;OOH and CE 18:2;OOH in CM and VLDL were mostly under LOQ. Therefore, it is expected that the highly sensitive MS will provide further information about lipoproteins oxidation in future studies. In conclusion, the insights about the oxidation mechanisms shown in this study would be useful for a more effective suppression of oxidative stress.

## Figures and Tables

**Table 1:** The ratio of PC 16:0/18:2;OOH to PC 16:0/18:2 and CE 18:2;OOH to CE 18:2.

	**PC 16:0/18:2 (µM)**	**The ratio of PC 16:0/18:2;OOH to PC 16:0/18:2 (×10^−3^%)**					**PC 16:0/18:2(10*E*,12*Z*);9OOH** **/PC 16:0/18:2(10*E*,12*E*);9OOH**
		**PC 16:0/18:2(10*E*,12*Z*);9OOH**	**PC 16:0/18:2(10*E*,12*E*);9OOH**	**PC 16:0/18:2;10OOH**	**PC 16:0/18:2;12OOH**	**PC 16:0/18:2(9*Z*,11*E*);13OOH** **+ PC 16:0/18:2(9*E*,11*E*);13OOH**	
CM (n = 9)	0.8	-	-	-	-	-	-
	0.3	-	-	-	-	-	-
	0.5	-	-	-	-	-	-
	3.6	-	-	-	-	58.4	-
	0.2	-	-	-	-	-	-
	1.1	-	-	-	-	10	-
	1.1	-	-	-	-	-	-
	0.9	-	-	-	-	-	-
	0.4	-	-	-	-	-	-
VLDL (n = 9)	5.7	-	-	-	-	13.4	-
	2.9	-	-	-	-	29.2	-
	5.8	-	-	-	-	42.3	-
	21.9	18	-	-	-	28.4	-
	4.5	-	-	-	-	25.6	-
	3.2	-	-	-	-	22.2	-
	14.9	18.5	-	-	-	27.3	-
	13.8	26	-	-	-	41	-
	6.5	-	-	-	-	27.2	-
LDL (n = 9)	27.8	9.4	-	-	-	14.8	-
	35.6	18.7	14.9	-	-	35.6	-
	22.9	12.8	-	-	-	23.6	-
	32.4	44	31.5	-	-	82.2	-
	28.7	10.8	-	-	-	20.4	-
	16.8	56.1	27.9	-	-	98.2	-
	41.7	75.2	44.1	0.9	0.7	138.5	-
	27.5	9	-	-	-	15.6	-
	31.7	58.8	33.4	-	-	112.3	-
	Mean ± SD(The ratio)	32.8 ± 25.8	-	-	-	60.1 ± 47.9	-
HDL (n = 9)	63.6	13.7	8.3	-	-	23.3	1.7
	45.1	68.7	33.9	0.8	0.7	120.5	2
	64.5	32.7	18.6	0.5	-	57.4	1.8
	28.7	93.7	47	1.3	1	168.3	2
	21.4	101.7	65.1	-	-	175.8	1.6
	32.7	19.4	10.9	-	-	34	1.8
	37.2	164.9	66.6	2.1	1.6	270	2.5
	39.2	78.1	45.5	0.9	0.6	135.1	1.7
	51.6	74.6	40.4	1	0.7	127.6	1.8
	Mean ± SD(The ratio)	71.9 ± 47.1	37.4 ± 21.5	-	-	123.6 ± 78.1	-
Significance (LDL vs. HDL)		<0.05	-	-	-	0.05	-
	**CE 18:2 (µM)**	**The ratio of CE 18:2;OOH to CE 18:2 (×10^−3^%)**					**CE 18:2(9*Z*,11*E*);13OOH** **/CE 18:2(9*E*,11*E*);13OOH**
		**CE 18:2(10*E*,12*Z*);9OOH** **+ CE 18:2(10*E*,12*E*);9OOH**	**CE 18:2;10OOH**	**CE 18:2;12OOH**	**CE 18:2(9*Z*,11*E*);13OOH**	**CE 18:2(9*E*,11*E*);13OOH**	
CM (n = 9)	16.8	-	-	-	10.1	-	-
	2.5	-	-	-	-	-	-
	4.5	-	-	-	-	-	-
	14.3	-	-	-	-	-	-
	3.7	-	-	-	-	-	-
	18.5	-	-	-	-	-	-
	17.6	-	1	-	13	-	-
	6.9	-	-	-	-	-	-
	4.4	-	-	-	-	-	-
VLDL (n = 9)	56.3	-	-	0.5	4.1	2	-
	15.4	-	-	-	11	-	-
	35.3	10.1	1	0.8	9.7	-	-
	213.9	8.3	0.5	0.4	8.3	0.6	-
	32.4	-	0.7	0.8	8.8	-	-
	21.2	-	-	-	-	-	-
	96.6	4.4	-	-	3.9	-	-
	126	3	-	-	2.1	0.9	-
	52.5	-	0.8	1	12.2	-	-
LDL (n = 9)	1738.2	3.2	1.6	0.1	6.6	0.3	22
	2131.1	2.9	0.6	0.2	12.5	0.5	25
	1185	3.1	0.3	0.3	12.9	0.7	18.4
	1860.5	3.8	0.1	0.1	4.1	0.3	13.7
	2017.5	3.9	0.1	0.1	4.4	0.8	5.5
	1204.1	6	0.2	0.3	7.2	1	7.2
	2783.5	6	0.1	0.3	10.4	0.8	13
	1710.2	5.3	0.2	0.2	6.3	1	6.3
	1877.6	6.7	0.2	0.3	8.4	1.2	7
	Mean ± SD(The ratio)	4.54 ± 1.45	0.38 ± 0.49	0.19 ± 0.09	8.09 ± 3.23	0.71 ± 0.32	-
HDL (n = 9)	1226.7	7.1	0.3	0.4	9.2	1.2	7.7
	1105.3	5	0.1	0.1	6.2	1	6.2
	1165.8	6.8	0.2	0.3	8.2	1.3	6.3
	480.3	7.9	0.3	0.5	11.5	1.8	6.4
	473	6	0.3	0.2	7	0.5	14
	644.8	7.2	0.4	0.3	7.8	0.7	11.1
	1017.1	6.6	0.2	0.2	7.5	1	7.5
	835.2	6	0.1	0.2	7.8	1.4	5.6
	1158.7	5.9	0.1	0.1	6.4	1.1	5.8
	Mean ± SD(The ratio)	6.50 ± 0.86	0.22 ± 0.09	0.27 ± 0.12	7.94 ± 1.63	1.11 ± 0.37	-
Significance (LDL vs. HDL)		<0.05	n.s.	n.s.	n.s.	<0.05	-

n.s. = not significance

## Data Availability

All of the data is contained within the article and the supplementary materials.

## Supplementary Materials

The following are available online at https://www.mdpi.com/article/10.3390/antiox10101598/s1, Supplementary Table S1: MS/MS parameters for detection of PC 16:0/18:2;OOH and CE 18:2;OOH isomers, Supplementary Table S2: MS parameters for detection of PC 16:0/18:2 and CE 18:2.

## Abbreviations

BHT	butylhydroxytoluene
CID	Collision-induced dissociation
EDTA	ethylenediaminetetraacetic acid
ESI	electrospray ionization
LOOH	lipid hydroperoxides
LOQ	limits of quantification
MxP	2-methoxypropene
MRM	multiple reaction monitoring
SPE	solid phase extraction

## References

[1] Kinoshita M., Oikawa S., Hayasaka K., Sekikawa A., Nagashima T., Toyota T., Miyazawa T. (2000). Age-related increases in plasma phosphatidylcholine hydroperoxide concentrations in control subjects and patients with hyperlipidemia. Clin. Chem..

[2] Chien C.-T., Chang W.-T., Chen H.-W., Wang T.-D., Liou S.-Y., Chen T.-J., Chang Y.-L., Lee Y.-T., Hsu S.-M. (2004). Ascorbate supplement reduces oxidative stress in dyslipidemic patients undergoing apheresis. Arterioscler. Thromb. Vasc. Biol..

[3] Adachi J., Matsushita S., Yoshioka N., Funae R., Fujita T., Higuchi S., Ueno Y. (2004). Plasma phosphatidylcholine hydroperoxide as a new marker of oxidative stress in alcoholic patients. J. Lipid Res..

[4] Nagashima T., Oikawa S., Hirayama Y., Tokita Y., Sekikawa A., Ishigaki Y., Yamada R., Miyazawa T. (2002). Increase of serum phosphatidylcholine hydroperoxide dependent on glycemic control in type 2 diabetic patients. Diabetes Res. Clin. Pract..

[5] Kato S., Nakagawa K., Suzuki Y., Asai A., Nagao M., Nagashima K., Oikawa S., Miyazawa T. (2015). Liquid chromatography–tandem mass spectrometry determination of human plasma 1-palmitoyl-2-hydroperoxyoctadecadienoyl-phosphatidylcholine isomers via promotion of sodium adduct formation. Anal. Biochem..

[6] Yamashita S., Kiko T., Fujiwara H., Hashimoto M., Nakagawa K., Kinoshita M., Furukawa K., Arai H., Miyazawa T. (2016). Alterations in the levels of amyloid-β, phospholipid hydroperoxide, and plasmalogen in the blood of patients with Alzheimer’s disease: Possible interactions between amyloid-β and these lipids. J. Alzheimers Dis..

[7] Yoshida Y., Umeno A., Shichiri M. (2013). Lipid peroxidation biomarkers for evaluating oxidative stress and assessing antioxidant capacity in vivo. J. Clin. Biochem. Nutr..

[8] Liebisch G., Fahy E., Aoki J., Dennis E.A., Durand T., Ejsing C.S., Fedorova M., Feussner I., Griffiths W.J., Köfeler H. (2020). Update on LIPID MAPS classification, nomenclature, and shorthand notation for MS-derived lipid structures. J. Lipid Res..

[9] Frankel E.N. (1985). Chemistry of free radical and singlet oxidation of lipids. Prog. Lipid Res..

[10] Ito J., Mizuochi S., Nakagawa K., Kato S., Miyazawa T. (2015). Tandem mass spectrometry analysis of linoleic and arachidonic acid hydroperoxides via promotion of alkali metal adduct formation. Anal. Chem..

[11] Shimizu N., Bersabe H., Ito J., Kato S., Towada R., Eitsuka T., Kuwahara S., Miyazawa T., Nakagawa K. (2017). Mass spectrometric discrimination of squalene monohydroperoxide isomers. J. Oleo Sci..

[12] Kato S., Nakagawa K., Suzuki Y., Suzuki K., Mizuochi S., Miyazawa T. (2014). Preparation of 13 or 9-hydroperoxy-9*Z*,11*E* (9*E*,11*E*) or 10*E*,12*Z* (10*E*,12*E*)-octadecadienoic phosphatidylcholine hydroperoxide. J. Oleo Sci..

[13] Kato S., Shimizu N., Ogura Y., Otoki Y., Ito J., Sakaino M., Sano T., Kuwahara S., Takekoshi S., Imagi J. (2021). Structural analysis of lipid hydroperoxides using mass spectrometry with alkali metals. J. Am. Soc. Mass Spectrom..

[14] Bartlett G.R. (1959). Phosphorus assay in column chromatography. J. Biol. Chem..

[15] Kato S., Shimizu N., Hanzawa Y., Otoki Y., Ito J., Kimura F., Takekoshi S., Sakaino M., Sano T., Eitsuka T. (2018). Determination of triacylglycerol oxidation mechanisms in canola oil using liquid chromatography–tandem mass spectrometry. NPJ Sci. Food.

[16] Itabe H., Yamamoto H., Imanaka T., Shimamura K., Uchiyama H., Kimura J., Sanaka T., Hata Y., Takano T. (1996). Sensitive detection of oxidatively modified low density lipoprotein using a monoclonal antibody. J. Lipid Res..

[17] Folch J., Lees M., Sloane-Stanley G.H. (1956). A simple method for the isolation and purification of total lipides from animal tissues. J. Biol. Chem..

[18] European Medicines Agency (2011). Guideline on Bioanalytical Method Validation.

[19] Milne G.L., Seal J.R., Havrilla C.M., Wijtmans M., Porter N.A. (2005). Identification and analysis of products formed from phospholipids in the free radical oxidation of human low density lipoproteins. J. Lipid Res..

[20] Shrestha R., Chiba H., Hui S.-P. (2020). Oxidized lipid species in lipoproteins: Significance and analytical considerations. Med. Mass Spectrom..

[21] MacMillan D.K., Murphy R.C. (1995). Analysis of lipid hydroperoxides and long-chain conjugated keto acids by negative ion electrospray mass spectrometry. J. Am. Soc. Mass Spectrom..

[22] Hong S., Lu Y., Yang R., Gotlinger K.H., Petasis N.P., Serhan C.N. (2007). Resolvin D1, protectin D1, and related docosahexaenoic acid-derived products: Analysis via electrospray/low energy tandem mass spectrometry based on spectra and fragmentation mechanisms. J. Am. Soc. Mass Spectrom..

[23] Derogis P.B.M.C., Freitas F.P., Marques A.S.F., Cunha D., Appolinario P.P., Paula F., Lourenço T.C., Murgu M., Mascio P.D., Medeiros M.H.G. (2013). The development of a specific and sensitive LC-MS-based method for the detection and quantification of hydroperoxy- and hydroxydocosahexaenoic acids as a tool for lipidomic analysis. PLoS ONE.

[24] Wheelan P., Zirrolli J.A., Murphy R.C. (1995). Analysis of hydroxy fatty acids as pentafluorobenzyl ester, trimethylsilyl ether derivatives by electron ionization gas chromatography/mass spectrometry. J. Am. Soc. Mass Spectrom..

[25] Milne G.L., Porter N.A. (2001). Separation and identification of phospholipid peroxidation products. Lipids.

[26] Seal J.R., Porter N.A. (2004). Liquid chromatography coordination ion-spray mass spectrometry (LC-CIS-MS) of docosahexaenoate ester hydroperoxides. Anal. Bioanal. Chem..

[27] Yin H., Porter N.A. (2007). Identification of intact lipid peroxides by Ag^+^ coordination ion-spray mass spectrometry (CIS-MS). Methods Enzymol..

[28] Ito J., Nakagawa K., Kato S., Hirokawa T., Kuwahara S., Nagai T., Miyazawa T. (2016). A novel chiral stationary phase HPLC-MS/MS method to discriminate between enzymatic oxidation and auto-oxidation of phosphatidylcholine. Anal. Bioanal. Chem..

[29] Brites F., Martin M., Guillas I., Kontush A. (2017). Antioxidative activity of high-density lipoprotein (HDL): Mechanistic insights into potential clinical benefit. BBA Clin..

[30] Nakagawa K., Kiko T., Miyazawa T., Burdeos G.C., Kimura F., Satoh A., Miyazawa T. (2011). Antioxidant effect of astaxanthin on phospholipid peroxidation in human erythrocytes. Br. J. Nutr..

[31] Vila A., Korytowski W., Girotti A.W. (2002). Spontaneous transfer of phospholipid and cholesterol hydroperoxides between cell membranes and low-density lipoprotein: Assessment of reaction kinetics and prooxidant effects. Biochem..

[32] Thiagarajan A.N., Chand P., Bhat B.V., Sridhar M.G. (2014). Assessment of oxidative stress in babies under phototherapy for neonatal jaundice. Int. J. Adv. Med. Health Res..

[33] Barclay L.R.C., Basque M.C., Stephenson V.C., Vinqvist M.R. (2003). Photooxidations initiated or sensitized by biological molecules: Singlet oxygen versus radical peroxidation in micelles and human blood plasma. Photochem. Photobiol..

[34] Karlsson H., Kontush A., James R.W. (2015). Functionality of HDL: Antioxidation and detoxifying effects. High Density Lipoproteins.

[35] Soran H., Schofield J.D., Durrington P.N. (2015). Antioxidant properties of HDL. Front. Pharmacol..

[36] Christison J.K., Rye K.A., Stocker R. (1995). Exchange of oxidized cholesteryl linoleate between LDL and HDL mediated by cholesteryl ester transfer protein. J. Lipid Res..

[37] Chowaniec Z., Skoczyńska A. (2018). Plasma lipid transfer proteins: The role of PLTP and CETP in atherogenesis. Adv. Clin. Exp. Med..

