# Supplementary material for: Investigation of Lipoproteins Oxidation Mechanisms by the Analysis of Lipid Hydroperoxide Isomers
Source: Antioxidants (Basel). 2021 Oct 12;10(10):1598. doi: 10.3390/antiox10101598 (PMC8533262; doi:10.3390/antiox10101598)
Supplement: Supplementary file 1 [file antioxidants-10-01598-s001.zip › antioxidants-1393889-supplementary.pdf]

Supplementary Table S1. MS/MS parameters for detection of PC 16:0/18:2;OOH and CE 18:2;OOH isomers.

	PC 16:0/18:2;9OOH	PC 16:0/18:2;10OOH	PC 16:0/18:2;12OOH	PC 16:0/18:2;13OOH	CE 18:2;9OOH	CE 18:2;10OOH	CE 18:2;12OOH	CE 18:2;13OOH
Polarity	ESI (+)				ESI (+)			
Precursor ion (*m/z*)	813 $[M+Na]^+$				704 $[M+Na]^+$			
Product ion (*m/z*)	388	684	683	541	195	576	575	247
Declustering potential (V)	145	145	145	145	130	141	91	106
Entrance potential (V)	10	10	10	10	13	10	10	10
Collision energy (V)	42	39	37	57	40	37	43	55
Collision cell exit potential (V)	20	17	17	23	10	30	16	14
Curtain gas (psi)	20				20			
Collision gas (psi)	4				4			
Ion spray voltage (V)	5500				5500			
Temperature (°C)	600				500			
Ion source gas 1 (psi)	40				40			
Ion source gas 2 (psi)	60				60			

Supplementary Table S2.
MS parameters for detection of PC 16:0/18:2 and CE 18:2

	PC 16:0/18:2	CE 18:2
Polarity	ESI (+)	ESI (+)
Mass range (*m/z*)	600-900	500-800
End plate offset (V)	500	500
Capillary (V)	3500	4100
Nebulizer (bar)	1.6	1.6
Dry gas (L/min)	6	6
Dry temp (°C)	180	180
Funnel 1RF (Vpp)	300	300
Funnel 2RF (Vpp)	400	400
Hexapole RF (Vpp)	400	400
isCID energy (eV)	0	0
Ion energy (eV)	10	10
Low mass (*m/z*)	200	300
Collision energy (eV)	15	18
Collision RF (Vpp)	750	850
Transfer time (µs)	45	35
Prepulse storage (µs)	10	13
